# Relationships between cognitive appraisal and roles/personality traits in basic life support

**DOI:** 10.20407/fmj.2021-008

**Published:** 2022-05-25

**Authors:** Tetsuya Nakamura, Sayuri Nakamura, Naoko Kageura, Akira Kondo, Yukika Hotta, Chikako Oda

**Affiliations:** 1 Department of Intensive Care Unit, Fujita Health University Hospital, Toyoake, Aichi, Japan; 2 Faculty of Nursing, Fujita Health University, School of Health Sciences, Toyoake, Aichi, Japan

**Keywords:** Basic life support, Nursing students, Cognitive appraisal, Controllability, Factor analysis

## Abstract

**Objective:**

To examine the relationship between the cognitive assessment of stress (cognitive appraisal) caused in a scenario requiring basic life support (BLS) and the roles during BLS/personality traits in nursing students.

**Methods:**

We conducted an anonymous self-administered questionnaire survey for 264 freshman and senior nursing students. The study period was one month from June 2019. The questionnaire included characteristics, roles (active involvement group/passive involvement group/no involvement group), Cognitive Appraisal Rating Scale (CARS), and Maudsley Personality Inventory (MPI). We only included data for female students (107 people) in the analysis because very little data is available for male students. The Mann-Whitney test was used for the comparison between two groups and the Kruskal-Wallis test was used for the comparison between three groups. The significance level was set at p<0.05.

**Results:**

The total number of responses was 133 (50.4%), and the number of valid responses was 107 (40.5%). As a result of analyzing the relationship between the role and the CARS subscale, the controllability of the active and passive involvement groups was significantly lower than that of the no involvement group (p=0.046). Also, the analysis of the relationship between the grade and the CARS subscale showed that the controllability was significantly lower in freshmen than seniors (p=0.020).

**Conclusion:**

This study showed the relationship between controllability and cognitive appraisal of stress in the simulation scenario of BLS. Therefore, it was suggested that support for improving controllability is necessary as a preventive measure to reduce the stress associated with BLS.

## 1. Introduction

The most common cause of death in the world is ischemic heart disease, accounting for approximately 16% of the total dealths.^1^ In Japan, which has entered a super-aging society, heart disease including ischemic heart disease is the second leading cause of death.^2^ Ischemic heart disease is the most common cause of sudden cardiac death, accounting for approximately 70% in Western countries and 25–50% in Japan.^3^ It develops suddenly and is more likely to result in a fatal situation. To increase the survival chance of sudden cardiac death from ischemic heart disease, basic life support (BLS) by citizens who happen to be present (bystanders) and medical professionals both inside and outside the hospital is urgently required. As the number of people suffering from ischemic heart disease has been increasing worldwide,^1^ the number of bystanders who perform BLS in such situations will also increase in the future. In a Japanese study among the patients who were witnessed at the time of cardiogenic cardiopulmonary arrest, the 1-month survival rate was 1.9 times higher in those who received BLS compared to those who did not receive BLS, suggesting the effectiveness of BLS.^4^

On the other hand, stress reactions that occur in some bystanders have been gaining attention in recent years.^5^

The stress theory advocated by Lazarus emphasizes the interaction between the individual and the environment. He stated that “stress is not a single variable, but a collective term for many variables and processes” and highlighted a strong influence of the individual cognitive process of evaluating stressors (cognitive appraisal) on differences in stress responses between individuals.^6^ Cognitive appraisal is defined as “cognitive processes that evaluate how stressful an individual-environment interaction is” and includes primary appraisal (commitment, appraisal for effect, appraisal for threat) and secondary appraisal (controllability).^7^ An effort to deal with what was evaluated as stressful is called coping.^6^

In the study by Mathiesen et al. interviewing 20 BLS bystanders, all of the study subjects responded that the experience affected their daily lives, and some reported stress responses.^8^ Also, in the Japanese study, 13 out of 18 bystanders reported stress responses.^9^ These findings suggest that the person involved in BLS goes through a stress reaction, which negatively affects daily life, despite dealing with the injured with good intentions based on humanitarian values.^8^ However, the long-term psychological effects of bystanders on out-of-hospital cardiac arrest (OHCA) are poorly understood, and further studies are needed.^5^ In terms of in-hospital cardiac arrest (IHCA), approximately 10% of acute care staff reported suffering from post-traumatic stress disorder (PTSD). Among them, those with fewer years of experience had the highest incidence of traumatic symptoms.^10^ Therefore, we need to consider preventive measures against stress reactions for everyone who performs BLS, including bystanders and medical professionals. There are not enough studies that investigated the specific factors causing stress reactions in performing BLS.

We developed a research question that the degree of cognitive stress appraisal may vary depending on the roles in BLS based on the previous qualitative research in Japan, reporting that the difference in BLS roles can be a factor that influences cognitive appraisal.^9^ We believe factor analysis to answer this research question is necessary because few previous studies have examined this matter in and out of Japan.

By understanding the relationship between the BLS roles and cognitive appraisal and identifying more stressful involvement in BLS, we can prioritize mental support for those roles to reduce stress reactions in BLS providers. In addition, by understanding the roles that are more likely to cause stress reactions during BLS in medical professionals, we can easily provide mental support based on the objective roles. Considering the difference in the way of perceiving stress (cognitive appraisal) depending on personality traits,^11^ we investigated the stress factors in a simulation scenario of BLS.

Based on the above, the purpose of this study is to examine the relationship between the cognitive appraisal and the roles during BLS/personality traits of nursing students in a BLS simulation scenario to establish preventive support.

## 2. Definition of Terms

1) Bystander: A general citizen who is present at the time of the incident

2) Cognitive appraisal: Subjective evaluation for the degree of stress in the interaction between the nursing student involved in BLS and the environment in BLS simulation scenario

## 3. Study Methods

### 1) Study Design

Anonymous self-administered questionnaire survey

### 2) Study Subjects

The study subjects were 264 nursing students, including 134 freshmen and 130 seniors at a nursing college, who received the information about this study from the researcher. Since the number of male data was extremely few, we only included female data in the analysis.

### 3) Survey Period

from mid-May 2019 to late-June 2019.

### 4) Survey Methods/Survey Contents

The survey form consists of (1) questions about characteristics, (2) questions about cognitive appraisal and roles, and (3) personality tests.

(1) Characteristics

Gender, grade, presence/absence/progress of BLS training, presence/absence of BLS experience and progress

(2) Cognitive Appraisal and Roles

① Cognitive Appraisal

The Cognitive Appraisal Rating Scale (CARS) was used to measure the cognitive appraisal. CARS was developed by Shinichi Suzuki et al..^12^ The reliability coefficient of CARS is α=0.52–0.84. For validity, the four factors consisting of the scale correspond to the elements of cognitive appraisal advocated by Lazarus & Folkman, appraisal for harmful/harmless, appraisal for threat, challenge, and controllability. The subscales are also generally consistent with the scales and subscales of existing cognitive appraisal measurements.^13^ Due to its reliability and validity, we selected CARS as a scale for cognitive appraisal in this study. For the scoring method, we calculated the total score of each subscale, “commitment (item 1, 2)”, “appraisal for effect (item 3, 4)”, “appraisal for threat (item 5, 6)”, and “controllability (item 6, 7)”. The score for each item was “strongly disagree”=0 points, “somewhat agree”=1 point, “agree”=2 points, and “strongly agree”=3 points (no reversal items). The score range for each factor was 0–6 points. A higher score means more active involvement in a stressor in “commitment”; more perception of harm to oneself in “appraisal for effect”; more perception of threat to a stressor in “appraisal for threat”; and more confidence to control the situation in “controllability”.^14^ “Commitment” and “controllability” reduce stress response while “appraisal for effect” and “appraisal for threat” increase stress response.^14^

* The following situation was presented as a simulation scenario.

“You are at the platform of the station early in the morning. Three people, including a man in his 70s, a woman in her 40s, and a woman in her 60s, are waiting for the train nearby. A few people are waiting for the train at the platform on the opposite side. You are using your smartphone while waiting for the train to depart 13 minutes later. Suddenly, the man in his 70s standing next to you collapses, and the three people on the platform are at the same distance (about 5 m) from him. When you observe him closely, he looks like he is snoring and sleeping.”

② Roles

Three roles during BLS were set for this study, and the students were asked to choose which role they would play.

• Active involvement group

This group is actively involved in patient care by approaching, calling 119 (ambulance), requesting an Automated External Defibrillator (AED), and performing chest compressions until an AED arrives.

The students who selected the active involvement group were asked to answer CARS while assuming that the following situation is currently happening to them:

“You approach the collapsed man and tap his shoulder, but he does not respond. You ask the women in their 40s and 60s to call 119 and bring an AED. You touch the carotid artery, but no pulse is present. Breathing and movement of the chest are absent when you place your ear close to his mouth and look at his chest. You determine it is cardiopulmonary arrest and start cardiopulmonary resuscitation. The skin of the patient is warm. Three minutes after starting cardiopulmonary resuscitation, an AED arrives. You ask to place the AED on the victim and suspend the chest compressions after the AED begins analyzing the rhythm. Looking around, you notice that a smartphone camera is pointed at you from the platform on the opposite side.”

• Passive involvement group

This group is not actively involved but passively involved in patient care according to the instructions.

The students who selected the passive involvement group were asked to answer CARS while assuming that the following situation is currently happening to them:

“You do not approach the man. A woman in her 40s standing on the other side of the collapsed man approaches and taps his shoulder, but he does not respond. The woman asks you to bring an AED. When you return with an AED, the woman in her 40s has already begun CPR. At that time, 3 minutes has passed since the man collapsed. While the woman performs the chest compressions, you place the AED on the man according to her instructions. The woman suspends the chest compressions as the AED begins analyzing the rhythms. Looking around, you notice that a smartphone camera is pointed at you from the platform on the opposite side.”

• No involvement group

This group is not involved in patient care while staying near the site of performing BLS.

The students who selected the no involvement group were asked to answer CARS while assuming that the following situation is currently happening to them:

“You move away from the scene immediately after the man collapsed, relying on someone else. A woman in her 40s standing on the other side of the collapsed man approaches and taps his shoulder, but he does not respond. A woman in her 60s calls 119 and goes looking for an AED. During that time, the woman in her 40s touches his neck and places her ear on his mouth. Shortly after, the woman in her 60s returns with an AED. At that time, 3 minutes has passed since the man collapsed. The woman in her 40s instructs the woman in her 60s to place the AED. She suspends the chest compressions as the AED begins analyzing the rhythms. You are staring at what is happening. Meanwhile, you notice that a smartphone camera is pointed at you from the platform on the opposite side.”

(3) Personality Traits

The Maudsley Personality Inventory (MPI) was used to measure the personality traits. The MPI was developed by Eysenck and organized by Jensen. It was translated to the Japanese version of MPI by the MPI Study Group.^15^ The reliability coefficient of MPI is as high as 0.84–0.90. The validity is also sufficiently proven with high factorial validity for the E (Extraversion/Introversion) and N (neuroticism) scales. We selected the MPI for this study due to its verified reliability and its validity. The MPI consists of 80 items, which are answered by “Yes”, “? (I cannot choose either)”, or “No”. The designated scoring board is placed on the answer sheet and used for scoring. Two points are given to each item designated for 2 points on the scoring board if you answer either “yes” or “no”, and 1 point is added to the designated item if you answer “?”. The scoring board is color-coded with blue for the N scale, red for the E scale, and black for the L scale. The MPI includes 20 lie-scale items called the L scale to detect the dishonesty of the subjects and how much they try to make themselves look better than they are. The possible range of scores is 0–48 points on the E and N scales and 0–40 points on the L scale. The scales are divided into a total of 9 classifications. The E scale is classified into E^−^ type (0–18 points) with more introversion, E^+^ type (30–48 points) with more extroversion, and E_0_ type (19–29 points) that does not belong to either. The N scale is classified into N^−^ type with less neuroticism (0–18 points), N^+^ with more neuroticism (30–48 points), and N_0_ type (19–29 points) that does not belong to either.

### 5) Analysis Methods

We used the Mann–Whitney U test for comparison between 2 groups (relationship between cognitive appraisal and characteristics) and the Kruskal-Wallis test for comparison between 3 groups (cognitive appraisal and roles during BLS/cognitive appraisal and personality traits). We performed the analyses using SPSS ver.22.0 with the statistical significance level at p<0.05.

### 6) Ethical considerations

This study was approved by the Ethics Review Board of the university to which the principal investigator belongs (Approval number HM18-522). We started to take the survey after obtaining permission from the Dean. We provided a written and oral explanation to the subjects about the study objectives, subjects, methods, ethical considerations, conflicts of interest, and contact information of the researchers. The survey was anonymous so that individuals could not be identified. Subjects were allowed to decide whether to participate in the study voluntarily. Posting the survey form in the collection box was considered as the consent to participate in the study. However, after posting the survey form, the decision to not participate in or discontinue the study was limited because this was an anonymous survey. We explained that declining participation or discontinuation would not cause any disadvantages. Due to the nature of the study, participants with an experience of BLS may have been psychologically affected. Therefore, we presented this risk during the study explanation and set the deadline for submission to one week from that day, giving sufficient time to consider whether to participate in the study. The survey forms posted in the collection box were stored in locked storage. The obtained information was processed by a computer equipped with antivirus software and saved in a USB memory with a password lock function, which was also stored in locked storage.

### 7) Conflict of Interest

Approval was obtained by the Conflict-of-Interest Committee of the university to which the principal investigator belongs (approval number CI18-610). There are no conflicts of interest to declare.

## 4. Results

The survey forms were collected from 133 out of 264 study subjects (response rate 50.4%). The responses with multiple missing data or the L scale ≧20 were excluded due to low credibility.^16^ The data for male students were also excluded due to the extremely small number of responses. As a result, the number of valid responses was 107 (valid response rate 40.5%).

### 1) Characteristics of Subjects

Table 1 shows the characteristics of the subjects. For the “grade”, 37 students were freshmen (34.6%), and 70 students were seniors (65.4%). For the “BLS training status”, 70 subjects (65.4%) previously attended, and 37 subjects (34.6%) never attended. Of the 70 subjects with previous BLS training, 19 subjects (27.1%) completed the training within the past 2 months, 7 subjects (10.0%) completed 2 months to 1 year ago, 18 subjects (25.8%) completed 1 to 2 years ago, and 19 subjects (27.1%) completed 2 to 3 years ago, and 7 subjects (10.0%) complete more than 3 years ago. Only two subjects (1.9%) answered that they had “experience of actually performing BLS”. Both responded they did not suffer from subsequent stress responses.

### 2) Role Selection

For the role selection, 70 people (65.4%) chose the “active involvement group”, 33 people (30.9%) chose the “passive involvement group”, and 4 people (3.7%) chose the “no involvement group”.

### 3) Cognitive Appraisal

Table 2 shows the mean and standard deviation of CARS in this study. The mean value of “commitment” was 4.51±1.40, the mean value of “appraisal for effect” was 3.82±1.77, the mean value of “appraisal for threat” was 1.79±1.73, and the mean value of “controllability” was 2.16±1.23. The mean and standard deviation for women in a previous study was 4.72±1.17 for “commitment”, 4.61±1.37 for “appraisal for effect”, 1.70±1.47 for “appraisal for threat”, and 2.90±1.33 for “controllability”.^12^ Compared to the previous study, the subjects of this study had lower scores in commitment, appraisal for effect, and controllability but higher scores in appraisal for threat.

### 4) Personality Traits

Table 3 shows the mean and standard deviation of the E/N scales in the subjects. The mean value of the E scale was 25.21±12.70. On the E scale, 33 subjects (30.8%) were E^−^ type with more introversion, 32 subjects (29.9%) were normal E_0_ type, and 42 subjects (39.3%) were E^+^ type with more extroversion. The mean value of the N scale was 25.55±11.66. On the N scale, 42 subjects (39.2%) were N^−^ type with less neuroticism, 19 subjects (17.8%) were normal N_0_ type, and 46 subjects (43.0%) were N^+^ type with more neuroticism. The mean and standard deviation was 26.30±10.36 for the E scale and 24.34±10.11 for the N scale in women in the previous study.^15^ Compared to the previous study, the score of the E scale was lower and the score of the N scale was higher in this study.

### 5) Relationships between characteristics, roles, or personality traits and cognitive appraisal

(1) Relationship between characteristics and cognitive appraisal

Table 4 shows the mean/standard deviation and significant difference of CARS for the characteristics. We examined differences between the grades for each subscale of CARS (commitment, appraisal for effect, appraisal for threat, and controllability) using the Mann-Whitney U test. The controllability score was significantly lower in freshmen than seniors (p=0.020). The Kruskal-Wallis test showed a significant difference between the groups divided based on the time of previous BLS training in the CARS subscale of controllability (p=0.023).

(2) Relationship between roles and cognitive appraisal

Table 5 shows the mean/standard deviation and significant difference of CARS for the roles. The Kruskal-Wallis test showed a significant difference in controllability among the active involvement group, the passive involvement group, and the no involvement group. The score was highest in the active involvement group, lower in the passive involvement group, and lowest in the no involvement group. (p=0.046). Furthermore, we examined the significance of the difference between the groups using the DANN test. However, no significant difference was observed between any groups after adjustment (p=0.053, n.s. for active involvement group and no involvement group, p=0.180, n.s. for passive involvement group and no involvement group, and p=0.874, n.s. for active involvement group and passive involvement group).

When the subjects were divided based on the grade (freshman and senior) and analyzed separately, there were no significant differences between the roles and CARS for any subscale in both freshmen and seniors.

(3) Relationship between personality traits and cognitive appraisal

Table 6 shows the mean/standard deviation and significant difference of CARS for personality traits. The Kruskal-Wallis test showed no significant difference between the personality traits for the E/N scales in each CARS subscale (E scale: commitment p=0.370, n.s., appraisal for effect p=0.706, n.s., appraisal for threat p=0.531, n.s., controllability p=0.465, n.s.) (N scale: commitment p=0.505, n.s., appraisal for effect p=0.630, n.s., appraisal for threat p=0.580, n.s., controllability p=0.175, n.s.).

## 5. Discussion

When we examined cognitive appraisal for different roles in a BLS scenario among nursing students in this study, significant differences were observed in controllability, which suggests a strong association between controllability and stress reactions.

The mean scores for controllability were 1.78±1.16 in freshmen and 2.36±1.23 in seniors, showing a significantly higher score in seniors. In the previous study, a higher controllability score was associated with a lower incidence of stress reactions while a lower controllability score was associated with a higher incidence of stress reactions.^17^ Therefore, the freshman was more likely to feel stress when simulating the situation where BLS is required. Also, in a 4-year longitudinal study on stress in 67 nursing students, controllability of cognitive appraisal was higher in seniors, which was proportional to the degree of stress and stress response.^18^ As a background of the higher controllability score in seniors, seniors may have acquired the ability to control their own emotions and adjust their behaviors to deal with stress from their experience compared to freshmen. The freshman in this study had just started studying nursing shortly after enrollment when taking the survey and had little medical knowledge and no practical experience. Since it was more difficult for freshmen to perform BLS based on medical grounds, they were closer to bystanders. On the other hand, seniors were closer to medical professionals because they had completed clinical training and had sufficient medical knowledge to perform BLS based on the medical grounds compared to the freshman. In this way, the subjects of this study were individuals who almost represent bystanders and medical professionals. Therefore, the significant difference between the grades in the controllability scores of this study suggests that bystanders without medical knowledge are more likely to perceive stress than medical professionals.

In this study, significant differences in controllability were observed between the different roles in the BLS scenario. The controllability score was the lowest in the active involvement group. The study results suggested that the situation requiring BLS may be more likely to be perceived as stress in the active involvement group, the passive involvement group, and the no involvement group in order.

Bystanders are worried about the outcome and may feel “it is their fault if the victim’s condition worsens”.^19^ Also, bystanders tend to establish a causal relationship between their BLS performance and the victim’s serious outcome including death, and not evaluate other factors contributing to the prognosis^8^. Therefore, bystanders may be especially obsessed with the victim’s outcome.

Controllability is the cognitive appraisal of how much stress a person can control^12^. Given that the involvement roles are associated with low controllability and obsession about the outcome, bystanders may not be able to cope with stress perceived during BLS effectively. It is highly possible that bystanders feel so responsible for their actions related to the victim’s life that they cannot cope with the stress appropriately. Therefore, it is important to emphasize that their involvement in BLS does not directly determine the victim’s prognosis because many factors including environmental factors are associated with the onset of cardiopulmonary arrest. Bystanders need to understand what is happening to the victim who requires BLS and other uncontrollable influential factors such as the ambulance arrival time or no shock indication of AED. It may be necessary to incorporate such backgrounds into BLS training as educational support. However, careful consideration is needed since understanding the internal factors of the victim may require medical knowledge and may raise further concerns when bystanders perform BLS.

Even medical professionals sometimes perceive stress by performing BLS because they also have preconceptions like bystanders. All nurses have a psychological burden in which they must understand the situation properly and quickly, make decisions, and take actions in sudden changes of the patient, possibly leading to death.^20^ Even nurses feel significant stress from the perception that their judgments and actions may directly link to the death of the patient and the fear of not being able to save the patient. If nurses can objectively evaluate their BLS performance and perceive the situation appropriately, they could reduce their psychological burden. Since medical professionals already have medical backgrounds, we believe that stress can be reduced by accurately recognizing the situation based on medical knowledge along with the educational support described above. The support for bystanders may also be useful for medical professionals.

The limitations of this study are the lack of incorporation of the impact of social desirability bias (SDB) into the study design and the limited examination of the relationship between differences in personality traits and cognitive appraisal.

SDB is a type of response bias that study subjects tend to answer questions in a manner desirable in their social position rather than reflecting their true feelings.^21^ In this study, some nursing students have chosen the role in BLS that is not their true preference but is desirable as a medical professional. In other words, some subjects may have chosen the active involvement role as a nursing student due to SDB. The unwilling choice of the active involvement role may have caused additional stress to appropriately deal with the situation in cognitive appraisal during BLS simulation, resulting in the low score of controllability in CARS. Therefore, a future study needs to incorporate SDB into the study design.

It is known that cognitive appraisal for stress varies depending on personality traits^11^, so this study incorporated the simple MPI into the test items. However, there were no significant relationships between the cognitive appraisal and the MPI scales of introversion, extroversion, and neuroticism. There is a previous study using the egogram that represents a more detailed ego state of personality traits. In the future, we need to examine the relationship between personality traits and cognitive appraisal using a scale further subdividing personality traits.

## 6. Conclusion

In this study that examined the relationships between cognitive appraisal and roles in the BLS simulation scenario/personality traits in nursing students, we found the following findings:

(1) The groups more involved in BLS tended to have lower controllability compared to the group not involved in BLS.

(2) The controllability was significantly lower in the freshman compared to the senior.

(3) No significant relationship was observed between personality traits and cognitive appraisal.

The study suggested that support for improving controllability is needed to prevent stress response associated with BLS.

## Figures and Tables

**Table1:** Characteristics of the subjects

Sex	Female	107 (100)

Grade (N=107)	Freshmen	37 (34.6)
	Seniors	70 (65.4)

Whether or not a person has taken Basic Life Support training (N=107)	No	37 (34.6)
	Yes	70 (65.4)

Progress since Basic Life Support training (N=70)	Less than 2 months	19 (27.1)
	2 months or more and less than 1 year	7 (10.0)
	1 year or more and less than 2 years	18 (25.8)
	2 years or more and less than 3 years	19 (27.1)
	More than 3 years	7 (10.0)

Experience in Basic Life Support (N=107)	No	105 (98.1)
	Yes	2 (1.9)

N(%), Mean±SD

**Table2:** Mean and standard deviation of CARS in this study

	Female(N=l 07)	
Commitment (Points)		4.51±l.40

	0 Points	1 (0.9)
	1 Points	1 (0.9)
	2 Points	6 (5.6)
	3 Points	20 (18.7)
	4 Points	22 (20.6)
	5 Points	20 (18.7)
	6 Points	37 (34.6)

Appraisal for effect (Points)		3.82±1.77

	0 Points	5 (4.7)
	1 Points	6 (5.6)
	2 Points	15 (14.0)
	3 Points	19 (17.8)
	4 Points	21 (19.6)
	5 Points	14 (13.1)
	6 Points	27 (25.2)

Appraisal for threat (Points)		1.79±1.73

	0 Points	33 (30.8)
	1 Points	15 (14.0)
	2 Points	34 (21.8)
	3 Points	8 (7.5)
	4 Points	6 (5.6)
	5 Points	5 (4.7)
	6 Points	6 (5.6)

Contro llability (Po ints)		2.16±1.23

	0 Points	9 (8.4)
	1 Points	22 (20.6)
	2 Points	38 (35.5)
	3 Points	23 (21.5)
	4 Points	12 (11.2)
	5 Points	2 (1.9)
	6 Points	1 (0.9)

N(%), Mean±SD

**Table3:** Mean and standard deviation of the E/N scales in the subjects

	Female (N=116)	
E scales (Points)		25.21±12.70

	Type E^–^ (0~18 Points)	33 (30.8)
	Type E_0_ (19~29 points)	32 (29.9)
	Type E^+^ (30~48 points)	42 (39.3)

N scales (Points)		25.55±11.66

	Type N^–^ (0~18 Points)	42 (39.2)
	Type N_0_ (19~29 Points)	19 (17.8)
	Type N^+^ (30~48 Points)	46 (43.0)

N(%), Mean±SD

**Table4:** Mean/standard deviation and significant difference of CARS for the characteristics

		N	Commitment	Appraisal for effect	Appraisal for threat	Controllability
Grade (N=107)	Freshmen	37	4.51±1.35	3.65±1.93	1.54±1.77	1.78±1.16
	Seniors	70	4.51±1.44	3.91±1.68	1.93±1.70	2.36±1.23

	*p*-value		0.871	0.592	0.220	0.020*

Whether or not a person has taken Basic Life Support training (N=107)	No	37	4.78±1.23	3.97±1.82	1.70±1.81	2.22±1.18
	Yes	70	4.37±1.48	3.74±1.75	1.84±1.69	2.13±1.26

	*p*-value		0.209	0.535	0.498	0.674

Progress since Basic Life Support training (N=70)	Less than 2 months	19	4.68±1.38	3.74±1.63	2.21±1.78	1.53±1.07
	2 months or more and less than 1 year	7	4.57±1.62	3.29±2.36	0.86±0.90	3.00±1.00
	1 year or more and less than 2 years	18	4.44±1.20	3.72±1.87	1.67±1.78	2.17±1.30
	2 years or more and less than 3 years	19	4.00±1.83	3.89±1.76	1.68±1.73	2.11±1.15
	More than 3 years	7	4.14±1.35	3.86±1.56	2.71±1.38	2.86±1.57

	*p*-value		0.785	0.982	0.123	0.023*

Experience in Basic Life Support (N=107)	No	105	4.49±1.40	3.82±1.78	1.79±1.74	2.14±1.24
	Yes	2	6.00±0.00	4.00±1.41	2.00±0.00	3.00±0.00

	*p*-value		0.121	0.972	0.622	0.248

Mean±SD *: *p*<0.05

**Table5:** Mean/standard deviation and significant difference of CARS for the roles

		N	Commitment	Appraisal for effect	Appraisal for threat	Controllability
Freshmen	Active involvement group	26	4.42±1.39	3.35±1.94	1.50±1.79	1.69±1.09
	Passive involvement group	11	4.73±1.27	4.36±1.80	1.64±1.80	2.00±1.34
	No involvement group	0	—	—	—	—

	*p*-value		0.594	0.111	0.820	0.436

Seniors	Active involvement group	44	4.59±1.47	4.18±1.72	2.11±1.90	2.20±1.17
	Passive involvement group	22	4.32±1.46	3.50±1.44	1.73±1.28	2.45±1.30
	No involvement group	4	4.75±1.26	3.25±2.36	1.00±1.16	3.50±1.00

	*p*-value		0.689	0.187	0.547	0.112

Total	Active involvement group	70	4.53±1.43	3.87±1.83	1.89±1.87	2.01±1.16
	Passive involvement group	33	4.45±1.39	3.79±1.60	1.70±1.45	2.30±1.31
	No involvement group	4	4.75±1.26	3.25±2.36	1.00±1.16	3.50±1.00

	*p*-value		0.917	0.829	0.687	0.046*

Mean±SD *: *p*<0.05

**Table6:** Mean/standard deviation and significant difference of CARS for personality traits

			N	Commitment	Appraisal for effect	Appraisal for threat	Controllability
Freshmen	E scales	Type E^–^	13	4.24±1.48	4.03±1.63	1.76±1.71	2.00±1.17
		Type E_0_	12	4.47±1.37	3.75±1.93	1.84±1.61	2.09±1.20
		Type E^+^	12	4.76±1.36	3.71±1.77	1.79±1.86	2.33±1.30

		*p*-value		0.410	0.660	0.514	0.379

	N scales	Type N^–^	7	4.50±1.52	3.74±1.70	1.67±1.69	2.36±1.28
		Type N_0_	10	4.89±1.33	3.63±2.06	2.05±2.07	2.21±1.32
		Type N^+^	20	4.37±1.32	3.98±1.73	1.80±1.63	1.96±1.13

		*p*-value		0.840	0.771	0.490	0.398

Seniors	E scales	Type E^–^	20	4.24±1.48	4.03±1.63	1.76±1.71	2.00±1.17
		Type E_0_	20	4.47±1.37	3.75±1.93	1.84±1.61	2.09±1.20
		Type E^+^	30	4.76±1.36	3.71±1.77	1.79±1.86	2.33±1.30

		*p*-value		0.305	0.446	0.884	0.550

	N scales	Type N^–^	35	4.50±1.52	3.74±1.70	1.67±1.69	2.36±1.28
		Type N_0_	9	4.89±1.33	3.63±2.06	2.05±2.07	2.21±1.32
		Type N^+^	26	4.37±1.32	3.98±1.73	1.80±1.63	1.96±1.13

		*p*-value		0.446	0.393	0.772	0.361

Total	E scales	Type E^–^	33	4.24±1.48	4.03±1.63	1.76±1.71	2.00±1.17
		Type E_0_	32	4.47±1.37	3.75±1.93	1.84±1.61	2.09±1.20
		Type E^+^	42	4.76±1.36	3.71±1.77	1.79±1.86	2.33±1.30

		*p*-value		0.370	0.706	0.531	0.465

	N scales	Type N^–^	42	4.50±1.52	3.74±1.70	1.67±1.69	2.36±1.28
		Type N_0_	19	4.89±1.33	3.63±2.06	2.05±2.07	2.21±1.32
		Type N^+^	46	4.37±1.32	3.98±1.73	1.80±1.63	1.96±1.13

		*p*-value		0.505	0.630	0.580	0.175

Mean±SD

## Supplementary Material

PDF-Japanese
