# Supplementary material for: Relationships between cognitive appraisal and roles/personality traits in basic life support
Source: Fujita Med J. 2022 May 25;9(1):22–9. doi: 10.20407/fmj.2021-008 (PMC9923453; doi:10.20407/fmj.2021-008)
Supplement: Supplementary file 1 — PDF-Japanese [file fmj-9-022-s001.pdf]

一次救命処置による認知的評価と役割・性格特性との関係

Tetsuya Nakamura, RN[1], Sayuri Nakamura, RN, PHN, PhD[2],Naoko Kageura,RN,CN,MSN[2],Akira Kondo,RN,MHS[2],Yukika Hotta,RN,PHN,MSN[2],Chikako Oda,RN,MSN[2]

[1]Department of Intensive Care Unit, Fujita Health University Hospital, Toyoake, Aichi, Japan,

[2]Faculty of Nursing, Fujita Health University School of Health Sciences, Toyoake, Aichi, Japan,

Original Article

Corresponding author : Sayuri Nakamura, RN, PHN, PhD
Faculty of Nursing, Fujita Health University School of Health Sciences, 1-98 Dengakugakubo, Kutsukake-cho, Toyoake, Aichi 470-1192, Japan
E-mail: sayuri@fujita-hu.ac.jp

アブストラクト

目的：BLS実施状況を想定した場面で生じる看護学生のストレスの認知的評価（以下、認知的評価）と、BLS実施時の役割・性格特性との関係を明らかにする。
方法：看護学生1・4年生264人を対象とし、無記名自記式質問調査法を用いて調査した。調査期間は、2019年6月から1カ月とした。調査内容は、基本属性、役割(積極的関与群・消極的関与群・無関与群)、認知的評価尺度(CARS)、モーズレイ性格検査(MPI)とした。男性のデータが極めて少数であったため女性のデータ(107人)を解析対象とした。2群間の比較はMann-Whitney U検定、3群間の比較はKruskal-Wallis検定で分析した。有意水準は$p < 0.05$とした。
結果：回収総数133部(50.4%)、有効回答数107部(40.5%)であった。役割とCARS下位尺度との関連を分析した結果、積極的関与群･消極的関与群のコントロール可能性が無関与群より有意に低いことが認められた($p=0.046$)。また、学年とCARS下位尺度との関連を分析した結果、1年生のコントロール可能性が4年生より有意に低いことが認められた($p = 0.020$)。
結論：本研究により、BLS実施状況に遭遇した場合を想定した時に生じる認知的評価としてコントロール可能性のストレス認知の関係が示唆された。したがって、BLS実施に伴うストレスの予防策として、コントロール可能性に働きかける支援が必要であることが示唆された。

キーワード：一次救命処置、看護学生、認知的評価、コントロール可能性、要因分析

1.序論

世界で最も多い死因は全体の約16%を占める虚血性心疾患(Ischemic heart disease)であり[1]、超高齢社会を迎えた我が国においても、虚血性心疾患を含む心疾患は死因の第2位を占めている[2]。虚血性心疾患は、心臓突然死の一番多い原因であり、欧米各国では約70%、日本では約25～50%を占めている[3]。そのため、突発的に発症し致死的な状況に至る可能性が高い。虚血性心疾患による心臓突然死の救命率を向上させるためには、病院内外問わず急変に居合わせた市民(以下、バイスタンダー)や医療者の一次救命処置 Basic Life Support（以下 BLS）が急務である。虚血性心疾患罹患者は世界的に増加傾向[1]にあり、それに伴い急変の場に居合わせ、BLSを実施するバイスタンダーも今後増加すると考えられる。我が国において、心原性心肺機能停止の時点が目撃された傷病者の1ヶ月後生存率は、BLSが実施されていない場合の約1.9倍救命効果が高くBLSの有用性は周知の通りである[4]。

その一方で、少数ではあるがバイスタンダーにストレス反応が発生することが近年注目されている[5]。

Lazarusが提唱するストレス理論では、個人と環境との相互作用を重視し、「ストレスは一変数ではなく、多くの変数、過程からなるものをまとめた総称である」と述べており、特定のストレス状況（ストレッサー）において表出されるストレス反応の個人差には、ストレッサーをどのように評価するかという個人の認知的な過程（Cognitive appraisal：認知的評価）が強い影響を及ぼしていることを強調している[6]。認知的評価は、「個人と環境との相互作用がどの程度ストレスフルであるかを評価する認知過程」と定義され、１次的評価（コミットメント、影響性の評価、脅威性の評価）と２次的評価（コントロール可能性）がある[7]。ストレスフルと評価したものに対応する努力をコーピングという[6]。

Mathiesenらは、BLSを実施したバイスタンダー20人に聞き取り調査したところ、研究対象者全員が日常生活に影響を与える経験だったと述べ、そのうちの数名にストレス反応が認められたと報告がある[8]。また、我が国においても、18例中13人にストレス反応が認められた報告がある[9]。人道主義的価値観に基づく善意で傷病者に対応したにも関わらず、対応した本人がBLSを実施したことによりストレス反応が生じ、日常生活に支障をきたすことが既に起きている[8]。しかし、院外心停止 Out of Hospital Cardiac Arrest（以下 OHCA）におけるバイスタンダーの長期心理学的影響はほとんど理解されておらず、更なる研究が必要であるとされている[5]。さらに、院内心停止 In Hospital Cardiac Arrest（以下 IHCA）に関わった結果、急性期治療スタッフの約10%が心的外傷後ストレス障害 Post Traumatic Stress Disorder（以下 PTSD）を生じ、経験年数の少ないスタッフがトラウマ症状を発症するリスクが最も高かったとの報告[10]もあり、バイスタンダーだけではなく医療者を含め、BLSを施行する全員に対しストレス反応への予防策を検討していく必要がある。

BLS 実施によってストレス反応の生じる具体的な要因については十分な調査が行われていない。

そこで、国内の質的研究[9]から、BLS 実施時の役割の違いにおいて認知的評価に影響を与える要因となり得る報告があることから、BLS 実施時の役割の違いにより、それぞれの役割でストレッサーとして認知する度合が異なるのではないかとリサーチクエスチョンを立てた。また、国内外で先行研究は少なく未だ十分な検討がされておらず、このリサーチクエスチョンによる要因分析の必要性は十分にあると考える。BLS 実施時の役割と認知的評価の関係を検証しストレッサーとして認知する度合が高い役割を明確にすることで、その役割に対し優先的に精神的なサポートすることができ、BLS 実施者のストレス反応を軽減することに繋がると考えられる。また、医療従事者がストレス反応を生じやすい BLS 実施時の役割を把握することで、客観的な役割の情報により精神的なサポートが容易となり、バイスタンダーをサポートする面からの利点があると考える。更に、性格特性によるストレスの捉え方(認知的評価)が異なること[11]を考慮し、BLS 実施状況に遭遇した場合を想定した時のストレス要因を検討する必要があると考えた。

以上より、本研究ではBLS実施状況を想定した場面に生じる看護学生の認知的評価と、BLS実施時の役割・性格特性との関係を明らかにすることを目的とし、予防的な支援のあり方を検討する。

2.用語の定義

1)バイスタンダー：急変に居合わせた一般市民

2)認知的評価： BLS 実施状況を想定した場面で、そこに関与する看護学生と環境との相互作用において、看護学生がどの程度ストレスであるかを主観的に評価すること。

3.研究方法

1)研究デザイン

無記名自記式質問紙調査

2)研究対象

研究者からの研究説明を行った看護系大学 1 年生 134 人、4 年生 130 人、合計 264 人を研究対象者とした。男性が極めて少数であったため女性のみを解析対象とした。

3)調査期間

2019 年 5 月中旬から 2019 年 6 月下旬まで。

4)調査方法・調査内容

調査用紙は(1)基本属性に関する設問(2)認知的評価と役割に関する設問(3)性格検査から構成される。

(1)基本属性

性別、学年、BLS 講習受講の有無･経過、BLS 経験の有無･経過･状況

(2)認知的評価と役割

①認知的評価

認知的評価を把握するため認知的評価尺度 Cognitive Appraisal Rating Scale(以下 CARS)を用いた。CARS は鈴木伸一らによって作成された[12]。CARS の信頼性はα係数＝0.52～0.84 である。妥当性について、尺度を構成する 4 つの因子の内容は、害―無害の評価、脅威の評価、チャレンジ、コントロール可能性という Lazarus＆Folkman が提唱した認知的評価の構成概念に対応しており、また、尺度を構成する下位概念は、既存の認知的評価を測定する尺度と下位尺度構成と概ね一致している[13]。以上から、信頼性と妥当性が検証された尺度であるため選択した。採点方法は、「コミットメント (項目 1、項目 2) 」、「影響性の評価 (項目 3、項目 4) 」「脅威性の評価 (項目 5、項目 6) 」「コントロール可能性 (項目 6、項目 7) 」に含まれる項目得点を合計して各下位尺度得点を算出する。各項目の得点は、「全く違う」＝0 点、「いくらかそうだ」＝1 点、「まあそうだ」＝2 点、「その通りだ」＝3 点とする (反転項目なし) 。各因子の得点範囲は 0 点～6 点となる。得点が高いほど、「コミットメント」はストレッサーに対して積極的に関与する傾向を、「影響性の評価」は自分に害が及ぶと捉える傾向を、「脅威性の評価」はストレッサーを脅威と捉える傾向を、「コントロール可能性」は状況をコントロールできると捉える傾向を意味し、ストレス反応を弱めるのは、コミットメントとコントロール可能性、強めるのは影響性の評価と脅威性の評価となる[14]。

※提示した事例は以下の通りである。

「あなたは朝早い時間に駅のホームにいます。近くには同じように電車を待つ人が 3 人おり、見た目は 70 代男性、40 代女性、60 代女性である。反対ホームにも電車を待っているが数人います。あなたは 13 分後に出発する電車を待って、スマートフォンを操作しています。すると突然、隣の人で電車を待っていた 70 代男性が倒れました。その人はあなたの 5m くらいの距離で倒れ、ホームにいる 3 人は同じ距離にいます。よく見るといびきをかいて寝ているように見えます。」

②役割

独自に BLS 実施時の役割を 3 つ設定し、自分ならどの役割を行うであろうか選択してもらった。

・積極的関与群

自ら傷病者に近づき 119 番通報や Automated External Defibrillator(以下、AED)の依頼、AED が届くまでの胸骨圧迫の実施など積極的に傷病者に関与

する群である。

積極的関与群を設定した人には、下記に示されている状況が、現在自分に起こっていると考え CARS に回答してもらった。

「あなたは、倒れた男性に近づいて肩を叩きながら呼びかけましたが反応はありません。近くにいた 40 代女性と 60 代女性に 119 番通報と AED を依頼しました。あなたは、頸動脈が触れないことを確認し、耳を口元に当て視線は胸元を見るが息はなく胸の動きもないことを確認しました。あなたは心肺停止と判断し、心肺蘇生を開始しました。胸骨圧迫時、倒れた人の皮膚は暖かく感じました。心肺蘇生を開始し 3 分経過した頃、AED が届きました。あなたは、AED を装着するように指示し、AED がリズムの解析を始めたため、胸骨圧迫を一時中断しました。周りを見渡すと、反対ホームからはスマートフォンのカメラが向けられていることに気づきました。」

・消極的関与群

自ら傷病者に関与せず指示されたために関与する群である。

消極的関与群を設定した人には、下記に示されている状況が、現在自分に起こっていると考え CARS に回答してもらった。

「あなたは、男性に近づきませんでした。倒れた男性を挟んであなたの反対側にいた 40 代女性がその男性に近づき、肩を叩きながら呼びかけたが反応はないようです。あなたは 40 代女性に AED を持って来るよう指示されました。AED を探し出し、AED を待って戻ると 40 代女性は心肺蘇生を開始していました。男性が倒れて 3 分が経過していました。40 代女性に AED を装着するよう指示されたため、胸骨圧迫が行われる中 AED を倒れた男性に装着しました。AED がリズムの解析を始めたため、40 代女性は胸骨圧迫を一時中断しました。周りを見渡すと、反対ホームからはスマートフォンのカメラが向けられていることに気づきました。」

・無関与群

BLSの現場近くにいるもののその場には関与しない群である。

無関与群を設定した人には、下記に示されている状況が、現在自分に起こっていると考え CARS に回答してもらった。

「あなたは男性が倒れた直後、自分以外に人がいたためその場から離れるように移動しました。倒れた男性を挟んであなたの反対側にいた 40 代女性がその男性に近づき、肩を叩きながら呼びかけたが反応はないようです。60 代女性が 119 番通報をし、AED を探しに行きました。その間、40 代女性は倒れた男性の首を触り、耳を口元に当てていました。間もなく心肺蘇生を開始しました。60 代女性が AED を探し出し、AED を待って戻って来ました。男性が倒れて 3 分が経過していました。40 代女性が 60 代女性に

AEDを装着するよう指示し、AEDがリズムの解析を始めたため40代女性は胸骨圧迫を一時中断しました。あなたは、自分が見入っていたことに気づくと同時に反対ホームからはスマートフォンのカメラが向けられていることに気づきました。」

(3)性格特性

性格特性を把握するためモーズレイ性格検査 Maudsley Personality Inventory(以下、MPI)用いた。MPI はアイゼンクが開発し、その後、ジェンセンによって編成され、MPI 研究会がジェンセンによって編成された MPI を訳編し日本版MPIが作成された[15]。MPIの信頼性係数は0.84〜0.90であり信頼性は高い。妥当性について、E(内向性−外向性尺度)･N (神経症的傾向尺度)両尺度は高い因子的妥当性を持つことが、十分に認められている。以上から、信頼性と妥当性が検証された尺度であるため選択した。項目は 80 項目あり、各項目は「はい」「？(どちらともいえない)」「いいえ」の 3 択である。採点方法は、所定の採点盤を検査用紙に重ね、それぞれの尺度項目の回答に対し採点盤の2の数字にあたる「はい」または「いいえ」と回答すれば2点、採点盤の１の数字にあたる「？(どちらともいえない)」と回答すれば 1 点が加算される。採点盤はN尺度が青、E尺度が赤、L尺度が黒と色分けされている。MPIには L 尺度という虚偽発見尺度 20 項目含まれており、被検者がどの程度自分を実際以上によく見せようとして回答しているかを調べる尺度であり、質問に対する不正直さを検出しようとするものである。配点について、E、N の得点は0〜48点、Lの得点は0〜40点の範囲である。分類は、合計9分類に分かれる。E 尺度は内向型が強い E^-型(0〜18 点)、外向性が強い E^+型(30〜48 点)、どちらにも該当しない E_0型(19〜29 点)に分類され、N 尺度は、神経症的傾向が弱い N^-型(0〜18 点)、神経症的傾向が強い N^+(30〜48 点)、どちらでもない N_0型(19〜29 点)に分類される。

5)分析方法

2群間の比較(認知的評価と基本属性の関係)は Mann‐Whitney U 検定、3群間の比較(認知的評価と BLS 実施状況を想定した時に選択する役割、認知的評価と性格特性)は Kruskal-Wallis 検定にて分析を行った。SPSS ver.22.0 を用いて行い、統計学的有意水準は $p<0.05$ とした。

6)倫理的配慮

研究責任者が所属する大学の医学研究倫理審査委員会にて承認(承認番号HM18-522)を得て実施した。調査は、学科長の許可を得てから開始した。対象者に対し、文書及び口頭により研究目的･対象･方法･倫理的配慮･利益相反･研究者の連絡先の説明を行った。調査用紙は無記名式とし、個人を特定できないよう

にした。対象者の研究参加への意思決定は対象者の自由意思で決定した。強制力が働かないよう、回収ボックスを設け投函をもって研究参加の同意を得られたものとした。ただし、無記名とするため調査用紙投函後はその個人の特定が不可能であることから、研究への不参加及び中断は調査用紙投函前までとした。本研究の不参加あるいは中断によって、不利益を被ることはないことを説明した。研究内容の特性上、実際に BLS の実施経験を有する人にとっては精神的な影響を受ける可能性がある。そのため、研究の説明時にこのリスクについて提示し、負担感を感じた場合は中断する余裕ができるよう、提出期限を研究説明から 1 週間とした。回収ボックスに投函された調査用紙は鍵のかかる保管庫に保管した。得られた情報はウイルス対策ソフトを常駐したパソコンで処理を行い、パスワードロック機能付きの USB メモリーに保存し、その USB メモリーは調査用紙と同様の鍵のかかる保管庫で保管した。

7)利益相反

研究責任者が所属する大学の利益相反委員会にて承認(承認番号 CI18-610)を得ている。本研究に開示すべき利益相反はない。

4. 結果

研究対象者 264 人に対し、133 人から調査用紙を回収した(回収率 50.4%)。そのうち、欠損が多いもの、または MPI において虚偽発見尺度である L 尺度が 20 点以上の者は信憑性が低いとされている[16]ため除外した。更に、男性は極めて少数であったため除外した。結果、有効回答は 107 人であった(有効回答率 40.5%)。

1)対象の基本属性

対象の基本属性を Table1 に示す。対象の「学年」は 1 年生が 37 人(34.6%)、4 年生が 70 人(65.4%)であった。「一次救命処置講習の受講状況」は、有りが 70 人(65.4%)、無しが 37 人(34.6%)であった。「一次救命処置講習の受講状況」のうち有りと答えた 70 人の中で「一次救命処置講習受講からの経過」は、2 ヶ月未満が 19 人(27.1%)、2 ヶ月以上 1 ヶ月未満が 7 人(10.0%)、1 年以上 2 年未満が 18 人(25.8%)、2 年以上 3 年未満が 19 人(27.1%)、3 年以上が 7 人(10.0%)であった。「一次救命処置を実際に実施した経験」が有ると答えた人は 2 人(1.9%)であった。その後のストレス反応は 2 人とも無しと回答した。

2)役割の選択

「積極的関与群」を選択した人は 70 人(65.4%)、「消極的関与群」を選択した人は 33 人(30.9%)、「無関与群」は 4 人(3.7%)であった。

3)認知的評価の実態

本研究の CARS 平均値･標準偏差を Table2 に示す。「コミットメント」の平均値は 4.51±1.40 点、「影響性の評価」の平均値は 3.82±1.77 点、「脅威性の評価」の

平均値は 1.79±1.73 点、「コントロール可能性」の平均値は 2.16±1.23 点であった。先行研究における女性の平均点・標準偏差は、「コミットメント」が 4.72±1.17、「影響性の評価」が 4.61±1.37、「脅威性の評価」が 1.70±1.47、「コントロール可能性」が 2.90±1.33 である。先行研究[12]と比較して、本研究の対象は、コミットメント、影響性の評価、コントロール可能性で得点が低く、脅威性の評価で得点が高い結果となった。

4)性格特性の実態

対象の E･N 尺度の平均値･標準偏差を Table3 に示す。E 尺度の平均値は 25.21±12.70 点であった。また、E 尺度において、内向性の強い E^-型は 33 人(30.8%)、普通の E_0型は 32 人(29.9%)、外向性の強い E^+型は 42 人(39.3%)であった。N 尺度の平均値は 25.55±11.66 点であった。また、N 尺度において、神経症的傾向の弱い N^-型は 42 人(39.2%)、普通の N_0型は 19 人(17.8%)、神経症的傾向の強い N^+型は 46 人(43.0%)であった。先行研究における女性の E･N 尺度の平均値･標準偏差は、E 尺度の平均値が 26.30±10.36、N 尺度の平均値が 24.34±10.11 である。先行研究[15]と比較して、本研究の E 尺度では得点が低く、N 尺度では得点が高い結果となった。

5)基本属性、役割、性格特性と認知的評価との関連

(1)基本属性と認知的評価との関係

基本属性における CARS の平均値･標準偏差と有意差を Table4 に示す。1 年生と 4 年生の各 CARS の下位尺度(コミットメント、影響性の評価、脅威性の評価、コントロール可能性)に差があるか Mann‐Whitney U 検定を行ったところ、コントロール可能性で 1 年生の得点が 4 年生に比較し有意に低かった(p＝0.020)。BLS 講習を受講したことのある群の中から各 BLS 講習受講経過の各 CARS の下位尺度に差があるか Kruskal-Wallis 検定を行ったところ、コントロール可能性で有意差が認められた(p＝0.023)。

(2)役割と認知的評価との関係

役割における CARS の平均値･標準偏差と有意差を Table5 に示す。積極的関与群と消極的関与群と無関与群の各 CARS の下位尺度に差があるか、Kruskal-Wallis 検定を行ったところコントロール可能性で有意差が認められ、積極的関与群、消極的関与群、無関与群の順にコントロール可能性の得点が低かった(p=0.046)。更に、どの群間に差があるか検証するため DANN 検定を行ったところ、各群間の調整済み有意確率は認めなかった(積極的関与群と無関与群：p=0.053,n.s.、消極的関与群と無関与群：p=0.180,n.s.、積極的関与群と消極的関与群：p=0.874,n.s.)。

1 年生と 4 年生を分け解析行ったところ、各学年とも役割と CARS との間に有意差のある下位尺度はなかった。

(3)性格特性と認知的評価との関係

性格特性における CARS の平均値･標準偏差と有意差を Table6 に示す。E･N 尺度の各 CARS の下位尺度に差があるか、Kruskal-Wallis 検定を行ったところ有意差はみられなかった(E 尺度：コミットメント；p＝0.370,n.s.、影響性の評価；p=0.706,n.s.、脅威性の評価；p=0.531,n.s.、コントロール可能性；p=0.465,n.s.)(N 尺度：コミットメント；p＝0.505,n.s.、影響性の評価；p=0.630,n.s.、脅威性の評価；p=0.580,n.s.、コントロール可能性；p=0.175,n.s.)。

5.考察

本研究において学年の違い及び BLS 実施状況を想定した場面の役割の違いよる認知的評価の検討の結果、コントロール可能性の得点に有意差が認められたことから、コントロール可能性が大きく関連している可能性があることが分かった。

各学年の平均値は、1 年生が 1.78±1.16 点、4 年生が 2.36±1.23 点であり 4 年生の方が高かった。先行研究では、コントロール可能性の得点が高い者はストレス反応の出現率が低く、逆にコントロール可能性の得点が低いものはストレス反応の出現率が高いといわれている[17]。したがって、4 年生と比較し 1 年生の方がコントロール可能性の得点が低いことから、1 年生の方が BLS 実施状況に遭遇した場合を想定した時にストレスを感じやすいと考えられる。4 年生の方がコントロール可能性の得点が高い背景として、看護学生 67 人を対象としたストレスに関する 4 年間の縦断的研究より、認知的評価であるコントロール可能性はストレス度、ストレス反応に比例して 4 年生が高くなっており[18]、ストレスへの対処として自らの情動や行動を調節して対処しようとする力が 1 年生より獲得できているためであると考えられる。本研究で対象とした 1 年生の状況として、入学間もなく看護学を学び始めた時期の調査であり、医学的な知識は乏少で実習経験もないことから、医学的根拠に基づき BLS を実施することが難しく、よりバイスタンダーに近い存在と捉えている。4 年生は、臨地実習を終え、医学的な知識が 1 年と比較し豊富にあると考えられることから、医学的根拠に基づき BLS を実施することができ、より医療者に近い存在と捉えている。以上より、バイスタンダー及び医療者に近い個人での検討が可能であると考え、本研究の研究対象者と設定している。したがって、学年とコントロール可能性の得点との間に認められた有意差より、医学的知識を持たないバイスタンダーは医療従事者と比較しコントロール可能性のストレス認知をしやすい可能性があることが示唆された。

本研究において、BLS 実施状況を想定した場面の役割の違いによりコントロール可能性に有意差が認められた。3 群の中で積極的関与群のコントロール可能性の得点が最も低く、積極的関与群、消極的関与群、無関与群の順に BLS 実施状況をストレスと認知しやすい可能性が示唆された。

バイスタンダーには、傷病者の予後に関する不安があり「傷病者の予後が悪くなるのは自分の責任」と捉えてしまうことが有る[19]。また、バイスタンダーは、自分の行ったBLSと傷病者の予後の間に因果関係を確立し、BLSを行ったことで重症または死亡に繋がったかどうかに関する懸念が生じ、傷病者の予後に寄与する他の要因を評価しない傾向がある[8]。したがって、バイスタンダーは特に傷病者の予後に対して執着してしまうことが考えられる。

コントロール可能性は、ストレス状況をどの程度コントロールできるかの認知的評価である[12]。BLS実施に関与することを想定した役割はコントロール可能性の得点が低いことと、バイスタンダーが傷病者の予後に執着することを鑑みると、傷病者の予後を意識するあまりコントロール可能性に関するストレス認知に対し、効果的なコーピングができないと考えられる。人の生死に関わる自分の行いに責任を感じ、ストレスに適切な対処をすることができずストレス反応が生じている可能性が高い。したがって、自分が関わった事と、傷病者の予後を直接的に意味づけるものではないことを強調する必要がある。なぜならば、傷病者が心肺停止に陥る要因はいくつも有り、また環境因子もいくつも有るためである。よって、BLSを実施する状況の傷病者には何が起こっているのか、また、バイスタンダーが制御できない救急車の到着時間や、AEDの非ショック適応など他の影響因子にどのようなものが有るのか、そうした背景を教育的支援としてBLS講習に組み込むことが必要であると考えられる。しかし、傷病者の内的要因は医学的な知識を要する場合があるため、バイスタンダーがBLSを実際に実施する際に懸念を増強させてしまう可能性があるため慎重な検討が必要である。

医療従事者であってもBLS実施によりストレスを感じてしまうことは、バイスタンダーのように、先入観に囚われた自動思考があると考えられる。看護師は、患者の死を感じる急変場面において、看護師として適切で迅速な状況把握や判断、行動が行えるかという不安を抱え、すべての看護師がそうした心理的負担を抱いている[20]。このことから、看護師においても、自分の判断や行動と患者の死に直接的に結び、更に自分の看護師という立場上、患者を救えなかったらという感情が加わることで大きなストレスとして残ることが予想できる。看護師が自分のBLSを客観的に評価し、適切に状況を認知することができれば、心理的負担を軽減できるであろう。なぜならば、医療従事者の場合は既に医学的な知識を持っているため、その知識と上記の教育的支援の内容を照らし合わせて状況を適切に認識することで、ストレスの軽減に繋がると考える。そのため、医療従事者に対する支援としても、バイスタンダーと同じ支援の活用は有用ではないかと考えられる。

研究の限界として、社会的望ましさのバイアス Social desirability bias（以下SDB）による影響への対応が研究デザインに組み込まれていないこと、性格特性の違いによる認知的評価との関係の検討が不十分である事が挙げられる。

SDBとは、研究対象者が自身の本当の気持ちを反映した反応を選択するのではなく、自身の社会的立場において望ましいと思われる反応を示す傾向を指す[21]。本研究対象者は、看護学生であるため、本当の自身の意に即した選択ではなく医療者として望ましい行動を選択した者が少なからず存在する。すなわち、SDBによる影響を受け、看護学生として積極的関与群を選ばざるを得ないと考えた対象者がいる可能性がある。そのため、BLS実施状況に遭遇した場面を想定した認知的評価に加え、本意ではない積極的関与群と回答したために状況を適切に対処できないストレスが生じ、CARSにおけるコントロール可能性の得点が低値となった可能性があることを示唆する。今後、SDBを考慮した研究デザインによる検討が必要である。

本研究では、性格特性によるストレスの捉え方(認知的評価)が異なること[11]を考慮し、簡便に検査できるMPIを検査項目へ組み込んだ。MPIは内向性と外向性、更には神経病的傾向の有無を評価する検査であるが、本研究ではMPIと認知的評価との間に有意な関係は見られなかった。先行研究では、性格特性をより細かい自我状態を表すエゴグラムを活用した報告がある。今後、性格特性をさらに細分化した尺度を用いることで認知的評価の関係を検討する必要があると考える。

6.結論

本研究で、看護学生を対象としてBLS場面を想定し認知的評価と役割・性格特性の関係を検討したところ以下のことが明らかになった。

①BLS実施に関与する群の方が関与しない群と比較し、コントロール可能性が低い傾向が確認された。

②1年生の方が4年生と比較し、コントロール可能性が有意に低かった。

③性格特性と認知的評価との間には有意な関係は確認されなかった。

したがって、BLS実施に伴うストレス予防対策としてコントロール可能性を高める援助が必要であることが示唆された。

7.謝辞

本研究にご協力いただきました皆様に感謝致します。

References

1.World Health Organization. Global Health Estimates 2019: Deaths by Cause, Age, Sex, by Country and by Region, 2000-2019;2020. <https://www.who.int/docs/default-source/gho-documents/global-health-estimates/ghe2019_cod_global_2000_20194e572f53-509f-4578-b01e-6370c65d9fc5.xlsx?sfvrsn=eaf8ca5_7> (Accessed January 18,2021).

2.Ministry of Health, Labour and Welfare. reiwa gan nen (2019) zinkou doutai toukei (kakuteisu) no gaiyou;2020. (in Japanese).< https://www.mhlw.go.jp/toukei/saikin/hw/jinkou/kakutei19/index.html>(Accessed January 18,2021).

3.Wong CX, Brown A, Lau DH, Chugh SS, Albert CM, Kalman JM, Sanders P. Epidemiology of Sudden Cardiac Death: Global and Regional Perspectives. Heart Lung Circ 2019;28(1):6-14.

4.Fire and Disaster Management Agency.reiwa 2 nenban syoubouhakusyo;2020.(in Japanese).<https://www.fdma.go.jp/publication/hakusho/r2/56707.html>(Accessed January 18,2021).

5.Mausz J, Snobelen P, Tavares W.. "Please. Don't. Die.": A Grounded Theory Study of Bystander Cardiopulmonary Resuscitation. Circ Cardiovasc Qual Outcomes 2018;11:e004035.

6. Lazarus R, Folkman S. Stress, Appraisal, and Coping. New York: Spring Publishing Company.1984.

7.Folkman S, Lazarus R S, Dunkel-Schetter C, DeLongis A, Gruen R J. Dynamics of a stressful encounter: Cognitive appraisal, coping, and encounter outcomes.Journal of Personality and Social Psychology.1986;992-1003.

8.Mathiesen WT,Bjørshol CA,Braut GS,Søreide E. Reactions and coping strategies in lay rescuers who have provided CPR to out-of-hospital cardiac arrest victims: a qualitative study.BMJ Open 2016;6:e010671.

9.Tajima N, Takahashi H, Hatanaka M, Aoki R, Inoue Y. Study about the mental stress of bystanders, initiated CPR. JJSEM 2013;16:656-65(in Japanese).

10.Spencer SA, Nolan JP, Osborn M, Georgiou A.. The presence of psychological trauma symptoms in resuscitation providers and an exploration of debriefing practices. Resuscitation 2019;142:175-81.

11.Shimada A, Ando M, Sugiura Y, Tokomiya H, Miyazima N.Kotonaru sutoresu jyoukyou ni oite seikakutokusei ga sutoresuninntitekihyouka ni ataeru eikyou kanngokei daigakusei wo taisyou tosita situmonnsityousa karano kenntou(Influence of Personality Characteristics on Stress Cognitive Assessment in Different Stress Situations: An Investigation from a Questionnaire Survey of Nursing University Students).Nihonkangogakaironbunsyu kangokyouiku 2011;41:150-53(in Japanese).

12.Suzuki S, Sakano Y.Development of a cognitive appraisal rating scale(CARS) and its validation. Waseda Human Science Research 1998;7:113-124(in Japanese).
13.Public Health Research Foundation. Stress scale guidebook.Tokyo:Jitsumukyouiku-shuppan;2008:187-91. (in Japanese)
14.Endo M,Matsuda E,Shibata R.Big five pa-sonaritei ga taijinsutoresuko-pingu ni oyobosu eikyou(Effects of Big Five Personality on Interpersonal Stress Coping). Bulletin of Edogawa University 2017;27:335-41(in japanese).
15.MPI kenkyukai.sin・seikakukensahou mo-zureiseikakukensa.Tokyo:Seisin shobo;1969:13-14,24-31,32,36-40,115-20,144-50,213-15.
16.Kishimoto Y,Imada H. Results of the administration of Maudsley Personality Inventory (MPI) to the Japanese university students.Jimbun ronkyu 1978;28:63-83(in Japanese).
17.Saigo T,Nakajima S,Ogawa S,Tayama J.Post-Traumatic Stress Symptoms of Disaster Medical Assistance Staff in the Great East Japan Earthquake: Relation to Controllability for Intrusion and Post-Traumatic Stress Symptoms. Japanese Journal of Behavioral Medicine 2013;19:3-10(in Japanese).
18.Ichimaru N,Yamamoto F,Noda J.Relationship among the Stress Self-rating Scale and the other Scales such as Stressors,Stress Responses and some Factors of Influence during Four on Nursing University Students.The journal of Tokyo Academy of Health Sciences 2001;4:77-82(in Japanese).
19.Tajima N,Hatanaka M. Regarding stress and coping method of BLS. Journal of Clinical and Experimental Medicine 2017;262:1109-13(in Japanese).
20.Kudo M,Kashiwagi K,Hashimoto H,Terao Y.Kyuhenbamen no kangoshi no sinri ya kodo no henka keikennensu ni yoru tigai(Changes in nurses' psychology and behavior in the event of sudden change: differences by years of experience).Naganoken kango kenkyu gakkai ronbunsyu 2014;34:73-75(in Japanese).
21.Grimm P.Social Desirability Bias. 2010; <https://doi.org/10.1002/9781444316568.wiem02057>(Accessed june 18,2021).

Table 1 Attributes of study participants.

		N(%),Mean±SD
Sex	Female	107(100)
Grade(N=107)	Grade1	37(34.6)
	Grade4	70(65.4)
Whether or not a person has taken Basic Life Support training(N=107)	No	37(34.6)
	Yes	70(65.4)
Progress since Basic Life Support training(N=70)	Less than 2 months	19(27.1)
	2 months or more and less than 1 year	7(10.0)
	1 year or more and less than 2 years	18(25.8)
	2 years or more and less than 3 years	19(27.1)
	More than 3 years	7(10.0)
Experience in Basic Life Support(N=107)	No	105(98.1)
	Yes	2(1.9)

Table 2 Mean and standard deviation of CARS.

		N(%),Mean±SD
		Female(N=107)
Commitment(Points)		4.51±1.40
	0 Points	1(0.9)
	1 Points	1(0.9)
	2 Points	6(5.6)
	3 Points	20(18.7)
	4 Points	22(20.6)
	5 Points	20(18.7)
	6 Points	37(34.6)
Appraisal for effect(Points)		3.82±1.77
	0 Points	5(4.7)
	1 Points	6(5.6)
	2 Points	15(14.0)
	3 Points	19(17.8)
	4 Points	21(19.6)
	5 Points	14(13.1)
	6 Points	27(25.2)
Appraisal for threat(Points)		1.79±1.73
	0 Points	33(30.8)
	1 Points	15(14.0)
	2 Points	34(21.8)
	3 Points	8(7.5)
	4 Points	6(5.6)
	5 Points	5(4.7)
	6 Points	6(5.6)
Controllability(Points)		2.16±1.23
	0 Points	9(8.4)
	1 Points	22(20.6)
	2 Points	38(35.5)
	3 Points	23(21.5)
	4 Points	12(11.2)
	5 Points	2(1.9)
	6 Points	1(0.9)

Table 3 Mean and standard deviation of E scales and N scales of Maudsley Personality Inventory.

N(%),Mean±SD

		Female(N=116)
E scales(Points)		25.21±12.70
	Type E^{-}(0～18Points)	33(30.8)
	Type E_{0}(19～29points)	32(29.9)
	Type E^{+}(30～48points)	42(39.3)
N scales(Points)		25.55±11.66
	Type N^{-}(0～18Points)	42(39.2)
	Type N_{0}(19～29Points)	19(17.8)
	Type N^{+}(30～48Points)	46(43.0)

Table 4 Mean, standard deviation and significant difference of attributes of study participants and psychological stress.

Mean±SD

		N	Commitment	Appraisal for effect	Appraisal for threat	Controllability
Grade(N=107)	Grade1	37	4.51±1.35	3.65±1.93	1.54±1.77	1.78±1.16
	Grade4	70	4.51±1.44	3.91±1.68	1.93±1.70	2.36±1.23
	p -value		0.871	0.592	0.220	0.020*
Whether or not a person has taken Basic Life Support training(N=107)	No	37	4.78±1.23	3.97±1.82	1.70±1.81	2.22±1.18
	Yes	70	4.37±1.48	3.74±1.75	1.84±1.69	2.13±1.26
	p -value		0.209	0.535	0.498	0.674
Progress since Basic Life Support training(N=70)	Less than 2 months	19	4.68±1.38	3.74±1.63	2.21±1.78	1.53±1.07
	2 months or more and less than 1 year	7	4.57±1.62	3.29±2.36	0.86±0.90	3.00±1.00
	1 year or more and less than 2 years	18	4.44±1.20	3.72±1.87	1.67±1.78	2.17±1.30
	2 years or more and less than 3 years	19	4.00±1.83	3.89±1.76	1.68±1.73	2.11±1.15
	More than 3 years	7	4.14±1.35	3.86±1.56	2.71±1.38	2.86±1.57
	p -value		0.785	0.982	0.123	0.023*
Experience in Basic Life Support(N=107)	No	105	4.49±1.40	3.82±1.78	1.79±1.74	2.14±1.24
	Yes	2	6.00±0.00	4.00±1.41	2.00±0.00	3.00±0.00
	p -value		0.121	0.972	0.622	0.248

* : p <0.05

Table 5 Means, standard deviations, and significant differences of roles and psychological stress.

Mean±SD

		N	Commitment	Appraisal for effect	Appraisal for threat	Controllability
Grade1	Positive involvement group	26	4.42±1.39	3.35±1.94	1.50±1.79	1.69±1.09
	Negative involvement group	11	4.73±1.27	4.36±1.80	1.64±1.80	2.00±1.34
	Uninvolved group	0	–	–	–	–
	p -value		0.594	0.111	0.820	0.436
Grade4	Positive involvement group	44	4.59±1.47	4.18±1.72	2.11±1.90	2.20±1.17
	Negative involvement group	22	4.32±1.46	3.50±1.44	1.73±1.28	2.45±1.30
	Uninvolved group	4	4.75±1.26	3.25±2.36	1.00±1.16	3.50±1.00
	p -value		0.689	0.187	0.547	0.112
Total	Positive involvement group	70	4.53±1.43	3.87±1.83	1.89±1.87	2.01±1.16
	Negative involvement group	33	4.45±1.39	3.79±1.60	1.70±1.45	2.30±1.31
	Uninvolved group	4	4.75±1.26	3.25±2.36	1.00±1.16	3.50±1.00
	p -value		0.917	0.829	0.687	0.046*

* : p<0.05

Table 6 Means, Standard Deviations and Significant Differences of Personality Traits and psychological stress.

Mean±SD

			N	Commitment	Appraisal for effect	Appraisal for threat	Controllability
Grade1	E scales	Type E^{-}	13	4.24±1.48	4.03±1.63	1.76±1.71	2.00±1.17
		Type E_{0}	12	4.47±1.37	3.75±1.93	1.84±1.61	2.09±1.20
		Type E^{+}	12	4.76±1.36	3.71±1.77	1.79±1.86	2.33±1.30
		p -value		0.410	0.660	0.514	0.379
	N scales	Type N^{-}	7	4.50±1.52	3.74±1.70	1.67±1.69	2.36±1.28
		Type N_{0}	10	4.89±1.33	3.63±2.06	2.05±2.07	2.21±1.32
		Type N^{+}	20	4.37±1.32	3.98±1.73	1.80±1.63	1.96±1.13
		p -value		0.840	0.771	0.490	0.398
Grade4	E scales	Type E^{-}	20	4.24±1.48	4.03±1.63	1.76±1.71	2.00±1.17
		Type E_{0}	20	4.47±1.37	3.75±1.93	1.84±1.61	2.09±1.20
		Type E^{+}	30	4.76±1.36	3.71±1.77	1.79±1.86	2.33±1.30
		p -value		0.305	0.446	0.884	0.550
	N scales	Type N^{-}	35	4.50±1.52	3.74±1.70	1.67±1.69	2.36±1.28
		Type N_{0}	9	4.89±1.33	3.63±2.06	2.05±2.07	2.21±1.32
		Type N^{+}	26	4.37±1.32	3.98±1.73	1.80±1.63	1.96±1.13
		p -value		0.446	0.393	0.772	0.361
Total	E scales	Type E^{-}	33	4.24±1.48	4.03±1.63	1.76±1.71	2.00±1.17
		Type E_{0}	32	4.47±1.37	3.75±1.93	1.84±1.61	2.09±1.20
		Type E^{+}	42	4.76±1.36	3.71±1.77	1.79±1.86	2.33±1.30
		p -value		0.370	0.706	0.531	0.465
	N scales	Type N^{-}	42	4.50±1.52	3.74±1.70	1.67±1.69	2.36±1.28
		Type N_{0}	19	4.89±1.33	3.63±2.06	2.05±2.07	2.21±1.32
		Type N^{+}	46	4.37±1.32	3.98±1.73	1.80±1.63	1.96±1.13
		p -value		0.505	0.630	0.580	0.175
